# Multifaceted implementation and sustainability of a protocol for prehospital anaesthesia: a retrospective analysis of 2115 patients from helicopter emergency medical services

**DOI:** 10.1186/s13049-023-01086-w

**Published:** 2023-04-30

**Authors:** Susanne Ångerman, Hetti Kirves, Jouni Nurmi

**Affiliations:** Department of Emergency Medicine and Services, Helsinki University Hospital and University of Helsinki, FinnHEMS 10, Vesikuja 9, 01530 Vantaa, Finland

**Keywords:** Prehospital emergency anaesthesia, Intubation, Implementation, Prehospital, On-scene time, HEMS

## Abstract

**Background:**

Prehospital emergency anaesthesia (PHEA) is a high-risk procedure. We developed a prehospital anaesthesia protocol for helicopter emergency medical services (HEMS) that standardises the process and involves ambulance crews as active team members to increase efficiency and patient safety. The aim of the current study was to evaluate this change and its sustainability in (i) on-scene time, (ii) intubation first-pass success rate, and (iii) protocol compliance after a multifaceted implementation process.

**Methods:**

The protocol was implemented in 2015 in a HEMS unit and collaborating emergency medical service systems. The implementation comprised dissemination of information, lectures, simulations, skill stations, academic detailing, and cognitive aids. The methods were tailored based on implementation science frameworks. Data from missions were gathered from mission databases and patient records.

**Results:**

During the study period (2012–2020), 2381 adults underwent PHEA. The implementation year was excluded; 656 patients were analysed before and 1459 patients after implementation of the protocol. Baseline characteristics and patient categories were similar. On-scene time was significantly redused after the implementation (median 32 [IQR 25–42] vs. 29 [IQR 21–39] minutes, *p* < 0.001). First pass success rate increased constantly during the follow-up period from 74.4% (95% CI 70.7–77.8%) to 97.6% (95% CI 96.7–98.3%), *p* = 0.0001. Use of mechanical ventilation increased from 70.6% (95% CI 67.0–73.9%) to 93.4% (95% CI 92.3–94.8%), *p* = 0.0001, and use of rocuronium increased from 86.4% (95% CI 83.6–88.9%) to 98.5% (95% CI 97.7–99.0%), respectively. Deterioration in compliance indicators was not observed.

**Conclusions:**

We concluded that clinical performance in PHEA can be significantly improved through multifaceted implementation strategies.

## Background

Airway management is an essential component of prehospital emergency care. Prehospital emergency anaesthesia (PHEA) with tracheal intubation is the most common advanced procedure in prehospital critical care [1]. Various teams perform this high-risk procedure under challenging conditions [2, 3]. Patients are potentially in need of urgent care in the hospital, and the rapid sequence intubation (RSI) procedure should not unduly prolong the prehospital phase [4]. To function optimally, the prehospital system must be well-coordinated and efficient [5]. Despite the time pressure, patient safety must be ensured [6].

Available data indicate that prehospital airway management is sterling in high-performing services, even when compared with in-hospital management [7, 8]. Good performance in prehospital airway management consists of preparation and implementation factors. Currently, the recommended approach includes the use of experienced operators, planning and training, a standardised protocol, limited drug choices, the use of the best possible equipment [9, 10]. The frequency of complications increases with repeated intubation attempts, and consequently, the first-pass success (FPS) rate is a vital quality indicator [2, 11].

Despite knowledge of best practices, changing clinical practice is difficult. A systematic review by Ebben found that adherence to guidelines and protocols ranges from 7.8% to 95% in the prehospital setting [12]. Several reasons for difficulties in adopting new methods have been identified, including inadequate guidelines, deficient awareness, lack of skills, and practical barriers such as unsuitable equipment or training resources [12]. Efficient implementation of new technologies to professionals requires a systematic approach and the application of quality indicators [13]. The science of multidisciplinary implementation addresses the complexity of getting health care providers to change their practice [14]. Recently, increasing attention has been paid to the sustainability of such changes in the long term after implementation [15].

We hypothesised that a systematic implementation of a comprehensive PHEA process in a helicopter emergency medical service (HEMS) unit and the whole collaborating emergency medical service (EMS) community would lead to constant improvement in on-scene time (OST) and FPS rate as well as the sustainability of key performance indicators of protocol compliance. We observed a change in the FPS rate shortly after implementation of the new laryngoscopy method included in the protocol [7]. After implementing the PHEA protocol, we carried out this before-and-after observational study with a five-year follow-up to evaluate the sustainability of the changes in clinical practice and to describe the exceptionally intensive implementation process.

## Methods

### Ethics

The study protocol was approved by the authorities of Helsinki University Hospital (§17 HUS/278/2018). Additional approval by an ethics committee was not required under Finnish legislation, as only anonymous registry data were collected, and the study had no effect on patient treatment. The change in clinical practice was to implement a prehospital anaesthesia protocol irrespective of data collection. The study did not affect patient treatment and therefore patient consent was not required nor acquired. The STROBE guidelines were followed in reporting the study [16].

### Study design

We performed a retrospective observational study in one HEMS unit. We analysed HEMS mission database data accomplished with airway registry and patient charts before (2012–2014) and after (2016–2020) implementation of the PHEA protocol. The implementation year (2015) was excluded from the analysis. The primary end-points were OST, FPS rate, and three protocol compliance indicators — the use of esketamine, rocuronium, and mechanical ventilation. We also included a description of the entirety of the PHEA protocol implementation in this study.

### Setting

The study was performed in a HEMS unit (FinnHEMS 10) serving a population of approximately 1.3 million in southern Finland over an area of 10,000 square km [17]. The collaborative ground EMS system consists of 100–120 ambulances and approximately 500 prehospital care professionals (advanced life support nurses and basic life support technicians) working for six different employers. The HEMS unit is also assisted by several rescue departments.

The HEMS unit is staffed by a three-member crew: a physician, a HEMS crew member, and a pilot. The physicians are mainly experienced specialists in anaesthesiology with postgraduate training in prehospital medicine. During the study period (2012–2020), 22 physicians were employed by the service. The HEMS crew members are firefighters or prehospital nurses with extensive training in aviation and prehospital critical care working exclusively in HEMS.

The emergency dispatchers dispatch the unit according to the predefined criteria, for example, major trauma, cardiac arrest, and unconsciousness. Also, the ambulance crews can request the HEMS response. The unit does not play a role in interfacility transfers. The HEMS unit is dispatched annually approximately 2500 times and performs prehospital anaesthesia about 250 times. Ambulance crews in the area do not perform tracheal intubation, except during a cardiac arrest.

**Fig. 1:**
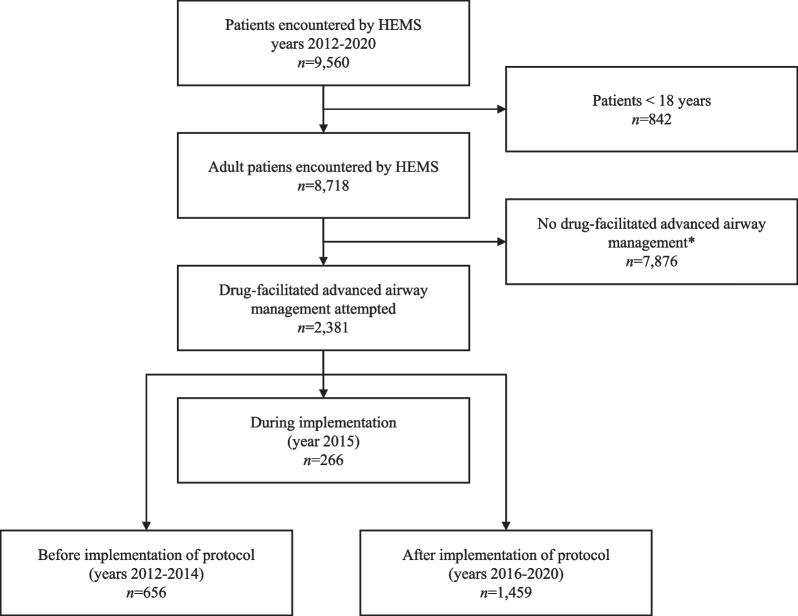
Flow chart of study population (2012–2020). *HEMS* helicopter emergency medical services. *Includes airway management without drugs, e.g. during cardiopulmonary resuscitation

The new protocol for PHEA was implemented in the HEMS unit and the collaborative EMS system at the beginning of 2015. The protocol consists of an inclusive description of the process, and it defines all actions from receiving the dispatch to handing over the patient at the hospital (Fig. 1). This protocol changed practice in several ways. The team is already organised with radio communication before the HEMS unit arrives to the patient. The EMS crews systematically prepare patients for induction of anaesthesia with a specific checklist while the HEMS unit is still en route. After arriving at the scene, the physician hears the report, examines the patient, and verifies the plan. The pre-anaesthesia checklist is read before induction.

Anaesthesia is primarily induced using esketamine, and a neuromuscular blocking agent (NMBA) (rocuronium > 1 mg kg^−1^) is mandatory. Use of fentanyl and propofol instead of esketamine is favoured in patients with status epilepticus, or markedly hypertensive patients with suspected intracranial haemorrhage, or isolated traumatic brain injury.

The intubation is always performed by the HEMS physician with a video laryngoscope (C-MAC pocket monitor, KARL STORZ Gmbh, Tuttlingen, Germany) and a bougie (Frova Intubating Introducer, 700 mm length, 3 mm diameter, no stiffening stylet, Cook Medical, Bloomington, USA), with the HEMS crew member assisting. The ambulance crew and sometimes rescue personnel are included in the assigned roles. Standard communication is used during laryngoscopy, and a pre-defined sequence is followed if visualisation of vocal cords is not achieved immediately. Because the routine use of a method combining video laryngoscopy and the Frova introducer was not described in earlier literature at the time of preparation of the protocol, we analysed the success rate shortly after implementation of the protocol to confirm the safety [7]. Mechanical ventilation is strongly favoured after intubation. Target values of vital signs and plans for treatment during transportation are defined aloud using the before-transportation checklist to ensure the whole team’s situational awareness.

Before the implementation of the PHEA protocol (2012–2014), preparation, drugs and laryngoscopy strategy used for prehospital intubation were not standardised. The physician on call determined the details individually. A traditional Macintosh laryngoscope and a stylet were the only equipment available in the years 2012–2013. In addition, the C-MAC video laryngoscope and the Frova introducer were available from the year 2014. During 2015, the conventional laryngoscope was removed, and only the C-MAC video laryngoscope was available. The use of NMBA was not mandatory and was based on clinical judgement. Succinylcholine and rocuronium were available, latter used usually at dose of 0.5 mg kg^−1^.

### Implementation of the protocol

The pre-implementation phase started during the preparation phase in autumn 2014, and implementation was accomplished during an intensive three-month period from January to March 2015. The implementation strategy was multifaceted, consisting of several methods and target populations (Table 1). The strategy and methods were planned after familiarising ourselves with applicable implementation frameworks and process models [14, 18–21]. Continuous support for protocol sustainability was offered after the implementation. The support consisted of identifying persistent deviations from protocol, regular on-duty training, and annual protocol development days at the HEMS base, simulation training with prehospital personnel, and scientific evaluation of the PHEA process.

**Table 1:** Implementation methods used to change clinical practice in prehospital anaesthesia

Implementation methods	Target population	Description	Timing
Preparation together with stakeholders	HEMS crew and representatives of EMS	Multifaceted workshops	3 months before implementation
Academic detailing	Physicians	One-on-one discussions aimed to overcome personal doubts	3 months before and during implementation
Education: skill station	Physicians, HEMS crew	On-duty training: new laryngoscopy method and standardised communication	1 month before and during implementation
Recognition of opinion leaders	Active members of EMS community	Open discussions aimed to encounter questions	1 month before and during implementation
Dissemination of information	EMS community, physicians, HEMS crew	RSI protocol and background material easily available and widely distributed	During and after implementation
Reminders	HEMS crew	Notes on key elements of the protocol placed in working environment	During implementation
Lectures	Prehospital nurses and technicians	20 lectures and discussion panels in the cooperation area	During and after implementation
Simulation	Physicians, HEMS crew, EMS personnel	Simulation training focusing on teamwork	During and after implementation
E-learning	EMS community	Public YouTube video	During and after implementation
Audit and feedback	Physicians	Observe deviations from protocol and discuss them	During and after implementation

### Inclusion criteria

The inclusion criterion for this study was drug-facilitated advanced airway management performed in adult patients (≥ 18 years) on the study units’ HEMS missions. Advanced airway management was defined as attempted tracheal intubation or surgical airway. Patients were divided into two groups: those treated in the three-year period (2012–2014) before implementation of the protocol and those treated in the five-year period (2016–2020) after its implementation. The implementation year (2015) was excluded from the analysis to avoid bias due to the sequential implementation process. The same general airway management indications existed throughout the study period, although they were made more visible to the prehospital community by the PHEA protocol*.*

### Data sources

The data were gathered primarily from a national HEMS mission database that has been used in Finland since 2012 [17, 22]. The physician on call enters the data into the database. The data consist of general alarm information, timestamps, patient characteristics and categorisation, and comprehensive records of procedures and treatment. From 2014, they also include structured prehospital airway data in accordance with recommendations for data gathering in prehospital settings [23]. The number of intubation attempts and dosage of induction agents in 2012 and 2013 had been reported in a structured way in patient charts, and these data were manually transferred to the study database for analysis.

### Outcome measurements

The OST was defined as the time from the HEMS unit reaching the scene to beginning their transportation (or leaving the scene, in cases in which the patient died on the scene). The OST was calculated from the timestamps in the database for the whole study period.

The FPS is defined as successful intubation on the first laryngoscopy attempt. The number of intubation attempts has been included in the structured HEMS patient record sheet for years before the launch of the national HEMS database. Since 2014, it has also been collected in the national HEMS database. Thus, FPS data were collected systematically during the study period.

The protocol compliance was evaluated through the use of esketamine and rocuronium in the induction of anaesthesia and the use of mechanical ventilation after intubation. The data concerning these drugs were collected from HEMS patient record sheets for 2012–2013 and from the database since 2014. The data on mechanical ventilation after intubation were available in the database for the whole study period.

### Statistical analyses

The normal distribution of continuous variables was tested using the D’Agostino and Pearson omnibus normality test. As virtually all parameters had a skewed distribution, we reported continuous variables as the median and interquartile range (IQR). The categorical parameters were compared between groups with Fisher’s exact test in cases of two categories and the chi-square test in cases of three or more categories. The continuous variables were compared with the Mann–Whitney U test. For proportions, 95% confidence intervals were calculated using the modified Wald method. The analyses were performed using GraphPad Prism, version 9.0.0, for Mac OS X (GraphPad Software, USA). A *p*-value of less than 0.05 was considered significant.

## Results

PHEA was performed by the HEMS unit on a total of 2381 adult patients during the study period (2012–2020). Data were analysed from 2115 patients after excluding 266 patients treated in the implementation year. Of the study patients, 656 (31.0%) were treated before implementation and 1459 (68.9%) after implementation of the protocol (Fig. 1). The baseline characteristics were similar (Table 2).

**Table 2:** Patient characteristics by year before and after implementation of the protocol

	2012 (*n* = 208)	2013 (*n* = 227)	2014 (*n* = 221)	2015 (*n* = 266)	2016 (*n* = 313)	2017 (*n* = 337)	2018 (*n* = 305)	2019 (*n* = 289)	2020 (*n* = 215)
Sex, male	135 (64.9)	150 (66.1)	141 (63.8)	165 (62.0)	206 (65.8)	218 (64.7)	201 (65.9)	194 (67.1)	152 (70.7)
Age, years	60 (44–68 [18–91])	55 (37–66 [18–87])	62 (41–73 [20–94])	57 (43–70 [19–97])	57 (37–68 [18–93])	53 (34–69 [18–90])	56 (36–69 [18–102])	54 (36–67 [18–95])	57 (40–70 [18–93])
HEMS unit reaching the patient, minutes from emergency call	37 (23–52 [10–125]), *n* = 195	33 (24–48 [10–108], *n* = 221	33 (23–49) [10–191]), *n* = 220	35 (25–50 [11–102]), *n* = 263	35 (24–52 [6–160]), *n* = 311	32 (23–44 [10–158]), *n* = 336	35 (25–47 [10–142]), *n* = 302	33 (23–47 [10–202]), *n* = 289	28 (21–46 [5–133), *n* = 215
*Patient categories*									
OHCA/post resuscitation	38 (18.3)	36 (15.9)	36 (16.3)	42 (15.8)	63 (20,.)	73 (21.7)	74 (24.3)	87 (30.1)	67 (31.2)
Trauma	41 (19.7)	54 (23.8)	52 (23.5)	72 (27.1)	76 (24.3)	56 (16.6)	66 (21.6)	58 (20.1)	50 (23.3)
Neurological	79 (38.0)	75 (33.0)	81 (36.7)	87 (32.7)	95 (30.4)	100 (29.7)	84 (27.5)	70 (24.2)	50 (23.3)
Intoxication	28 (13.5)	36 (15.9)	35 (15.8)	40 (15.0)	49 (15.7)	94 (27.9)	63 (20.7)	56 (19.4)	37 (17.2)
Other	22 (10.6)	26 (36.6)	17 (7.7)	25 (9.4)	30 (9.6)	14 (4.2)	18 (5.9)	18 (6.2)	11 (5.1)
*Vital signs upon HEMS crew arrival to the patient*									
Heart rate (beats.min)	91 (70–118 [30–170]), *n* = 164	100 (76–120 [10–200]), *n* = 163	91 (75–110 [30–176]), *n* = 205	98 (79–114 [20–188]), *n* = 251	93 (76–114 [28–180]), *n* = 283	94 (73–113 [20–171]), *n* = 312	94 (76–113 [40–195]), *n* = 280	96 (75–116 [32–197]), *n* = 252	96 (76–120 [30–170]), *n* = 189
Systolic blood pressure (mmHg)	130 (110–160 [46–250]), *n* = 159	133 (112–162 [44–240]), *n* = 162	134 (113–171 [60–250]), *n* = 202	134 (114–166 [65–253]), *n* = 245	136 (115–166 [45–262]), *n* = 276	135 (112–161 [25–258]), *n* = 305	132 (109–155 [46–241]), *n* = 274	134 (114–162 [50–264]), *n* = 245	135 (112–162 [49–266]), *n* = 181
Oxygen saturation (%)	97 (92–100 [30–100]), *n* = 148	95 (91–99 [50–100]), *n* = 151	96 (91–99 [21–100]), *n* = 197	97 (93–98 [46–100]), *n* = 241	97 (92–99 [18–100]), *n* = 272	97 (94–99 [55–100]), *n* = 299	98 (95–99 [50–100]), *n* = 273	98 (94–100 [23–100)], *n* = 238	97 (93–99 [24–100]), *n* = 183
Glascow coma score	3 (3–5 [3–15]), *n* = 195	4 (3–7 [3–15]), *n* = 194	4 (3–6 [3–15]), *n* = 219	3 (3–6 [3–15]), *n* = 265	4 (3–7 [3–15]), *n* = 307	3 (3–6 [3–15]), *n* = 336	3 (3–6 [3–15]), *n* = 298	3 (3–6 [3–15]), *n* = 283	3 (3–6 [3–15]), *n* = 213

Data are presented as values (%) or as median (interquartile range, [range]). Data available on every patient unless otherwise stated

**Fig. 2:**
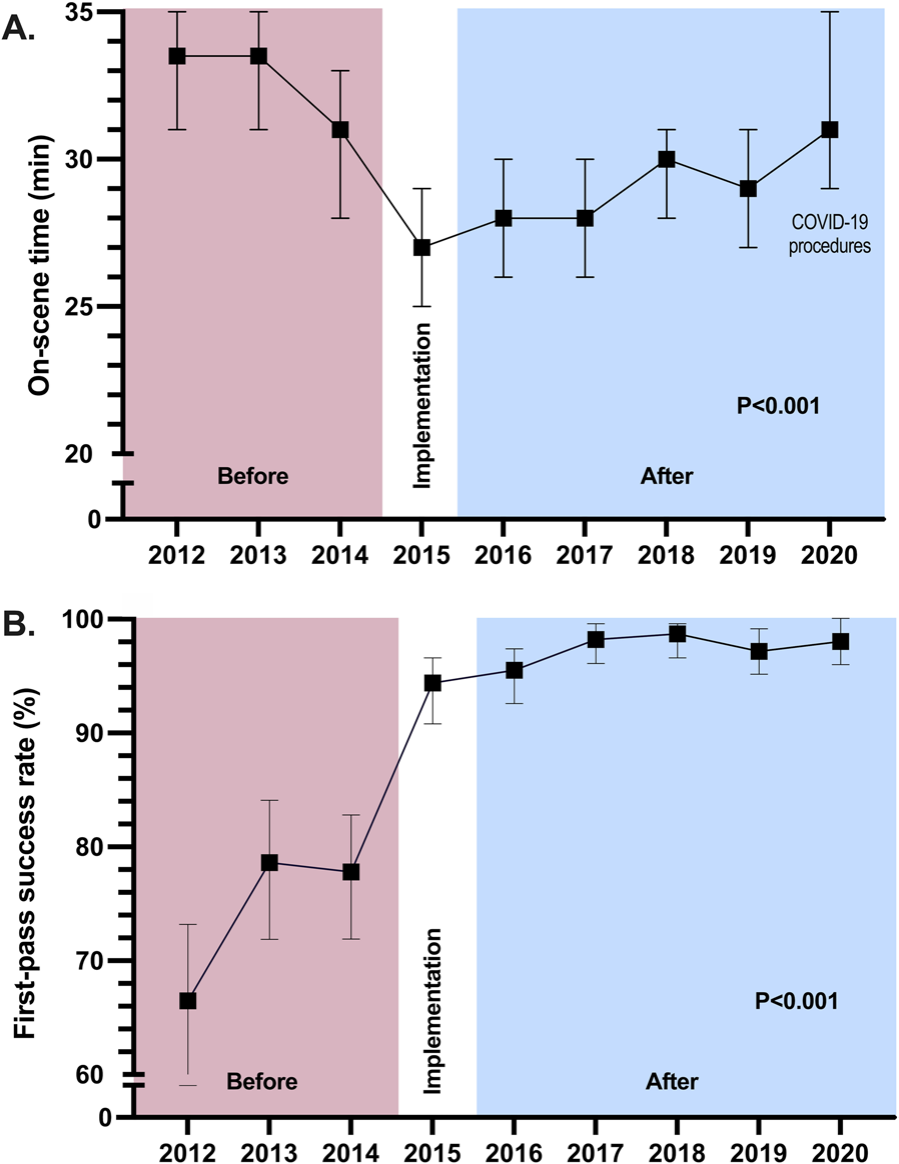
Progress of **A** on-scene time and **B** first-pass success rate before, during, and after implementation of protocol. On-scene time is presented as annual median with interquartile range and first-pass success rate as a percentage with 95% CI

The OST was significantly shorter after implementation of the protocol. The median OST was (32 [25–42] vs. 29 [21–39] minutes, a difference of − 3 [95% CI − 2 to − 4], *p* < 0.001. The change in OST remained throughout the follow-up period (Fig. 2A). Year 2020 was exceptional due to the COVID-19 protection procedures that caused OST to increase slightly. Excluding 2020, the change in OST was − 4 (95% CI − 5 to − 3) minutes. Data for calculating on-scene time were available in 617 cases in the pre-implementation group and 1404 in the post-implementation group.

Before the implementation, intubation was successful at the first attempt in 427 patients (74.4% [95% CI 70.7–77.8%]) and after protocol deployment in 1424 patients (97.6% [95% CI 96.7–98.3%]), *p* = 0.0001. Data on intubation attempts were available for 575 of 656 patients before the implementation and in all patients after. The progress of the FPS rate over nine years is shown in Fig. 2B.

**Fig. 3:**
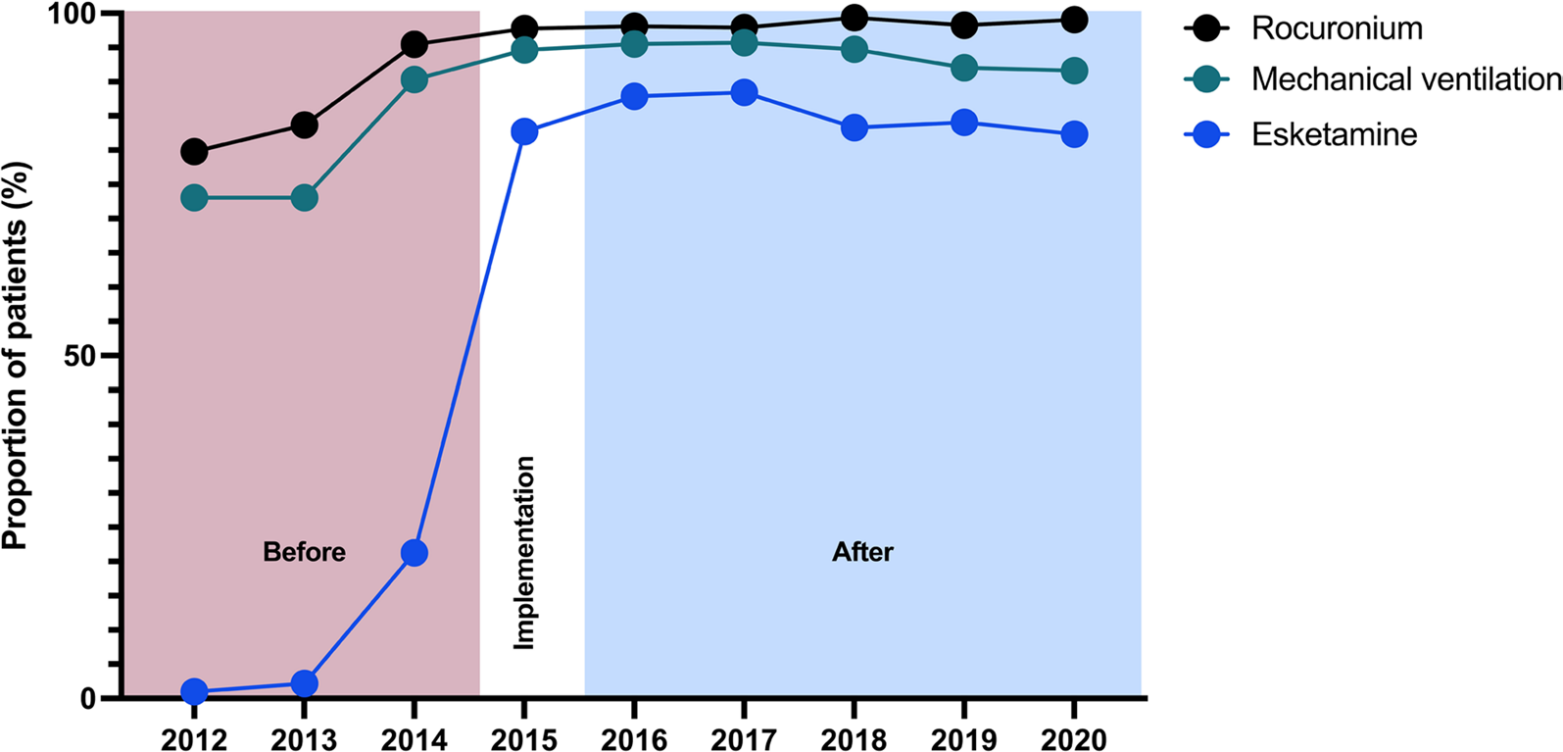
Progress of protocol compliance indicators during the study period

The key indicators for protocol compliance are presented in Fig. 3. The utilisation of mechanical ventilation increased from 70.6% (67.0–73.9%) to 93.4% (92.3–94.8%), *p* = 0.0001, during the study period. The use of rocuronium increased from 86.4% (83.6–88.9%) to 98.5% (97.7–99.0%) and of esketamine from 8.2% (6.3–10.6%) to 85.5% (83.6–87.2%), *p* = 0.0001 for both. The protocol compliance indicator data were available for the whole study population.

## Discussion

We found that the multifaceted implementation of the protocol for PHEA throughout the EMS led to shorter on-scene time, improved FPS rate, and high protocol compliance. The changes remained throughout the five-year follow-up period.

The quality and safety of PHEA and airway management have been increasingly emphasised in the last decade [24]. Recent studies have demonstrated that using standardised methods and the best tools recognised for emergency airway management improves results [2, 25]. However, the long-term sustainability of the improvement has not been previously reported.

A prolonged OST is associated with increased mortality [4, 26]. Prehospital OST is highly variable depending, for example, on logistical delays, system-related factors, and the level of care on scene [1, 5]. PHEA can be safely and effectively performed without significant delay in transport [27, 28]. Based on our finding of the shortened OST, it seems that the robust structure of the PHEA process enhanced the performance of the team, leading to better efficacy without compromising patient safety. The protocol involves EMS personnel as active team members not only during PHEA, but even before HEMS crew arrival as a result of a well organised team. Notably, no fewer than three checklists are used during the process. The protocol chanced practice in many ways and separating mechanisms leading to shorter OST is impossible. We consider that the active participation and training of EMS crew members in the implementation phase leads to widespread commitment to the protocol, with a positive impact on the process.

Targeting for a high FPS rate in prehospital airway management reduces severe complications related to repeated intubation attempts [2, 29]. The conditions for the first intubation attempt are optimised by pre-induction procedures, such as determination of the place for intubation, allocation of roles, thorough pre-oxygenation, head positioning, anticipation of the common challenges, and by the use of a fast-acting sedative and NMBAs [30]. The operator experience affects the FPS rate of intubation and the risk of physiological deterioration [9]. We implemented a bundle of interventions simultaneously and thus cannot separate the effects of individual components to FPS. However, we reported bigger improvement in FPS than most studies comparing videolaryngoscopy with direct laryngoscopy [31, 32]. This may suggest that a structured implementation plan focusing on multiprofessional teamwork and providing the best possible equipment are essential to achieve a high FPS rate.

Adherence to guidelines and protocols has been reported to range from 7.8 to 95% in the prehospital environment and from 0 to 98% in the emergency department setting [12]. Implementation of new technologies is complex, and there is a paucity of evidence of optimal implementation methods in the prehospital field [33]. In general, a successful change in clinical practice requires identification of barriers, multifaceted implementation strategies, and continuous training and monitoring [13, 33]. For example, Adelgais et al. described how organisational and technical barriers impeded implementation of prehospital pain management guidelines [35]. Motivation of personnel and intensive education are associated with successful implementation [36].

Implementation science is a scientific approach to promote the systematic uptake of evidence-based practices into routine practice to improve the quality and effectiveness of healthcare [14]. There are three overarching goals: guiding the process, understanding what influences implementation, and evaluating implementation [14]. Identifying the nature of the process to be implemented and choosing a suitable framework or combination of models are critical [37, 38]. We tailored a combination of ten different actions to enhance awareness, knowledge, and skills as well as to modify attitudes of individual HEMS professionals and hundreds of paramedics during the implementation (Table 1).

Sustainability is defined as the routine use of process components at sufficient intensity for the continuous achievement of desired goals and outcomes [15]. This is specified as a retrospectively viewed “outcome” in which health benefits or activities are maintained but also as a prospectively viewed “process” in which adaptation, learning, and continuous development are important for continuation and maintenance of a desirable feature [15]. Sustainability is a relatively young and complex concept in healthcare [39]. It has been measured after implementation in various heathcare fields [40, 41]. OST, FPS, and protocol compliance seem to be suitable “outcomes” for PHEA protocol sustainability. In the current study, all these remained high throughout the follow-up period and even showed some improvement in later years. Based on our experience, the high sustainability of a critical process is reached through continuous efforts in education, development, and auditing. This should be recognised when planning the introduction of new treatments and guidelines.

A major strength of this study is its robust data source. However, the data collection was partly retrospective and prospective data collection was not carried out in purposes of the current study. Self-reporting bias should be considered, but experience in structured database input and utilisation for over twenty years favour decreased risk of this bias. Other strengths include a relatively large number of patients and a long follow-up period. The main limitation of this study is that the protocol was implemented and data collected only in one HEMS unit, and generalisability may be limited. It is unknown whether the study unit or the culture of the collaborating EMS have features favouring success with these methods. However, the methods used in implementation need to be tailored to the system, and multi-centre studies of multifaceted implementation are therefore challenging. Furthermore, the study indicated favourable outcomes as a result of bundling actions, but specific key interventions cannot be pointed out.

The demands for both efficiency and safety are high in many healthcare systems and they may be seen as competing interests. In our experience, procedures to improve patient safety can be implemented without causing delays in a time-critical process. Our experience with the multifaceted implementation of a protocol for PHEA can be utilised as such in several in-hospital fields. Effective implementation and a high level of sustainability require great effort and consume resources. Thus we recommend careful allocation of the limited resources to the most valuable and evidence-based technologies and processes. We also encourage organisations to choose sustainability indicators for the assessment of valuable processes.

## Conclusions

A high level of patient safety and efficacy in PHEA can be achieved and sustained using multi-faceted interventions spread across organisation boundaries. The workload of the interventions is high, and thus, the clinical processes to be addressed should be carefully prioritised and selected.

## Data Availability

The dataset analysed during the current study is available from the corresponding author on reasonable request.

## Abbreviations

CI: Confidence interval
EMS: Emergency medical service
FPS: First-pass success
HCM: HEMS crew member
HEMS: Helicopter emergency medical service
IQR: Interquartile range
NMBA: Neuromuscular blocking agent
OST: On-scene time
PHEA: Prehospital emergency anaesthesia
STROBE: Strengthening the reporting of observational studies in epidemiology
